# Genotoxicity of Particles From Grinded Plastic Items in Caco-2 and HepG2 Cells

**DOI:** 10.3389/fpubh.2022.906430

**Published:** 2022-07-06

**Authors:** Martin Roursgaard, Monika Hezareh Rothmann, Juliane Schulte, Ioanna Karadimou, Elena Marinelli, Peter Møller

**Affiliations:** Section of Environmental Health, Department of Public Health, University of Copenhagen, Copenhagen, Denmark

**Keywords:** nanoparticles, microplastic, oxidative stress, DNA damage, comet assay

## Abstract

Large plastic litters degrade in the environment to micro- and nanoplastics, which may then enter the food chain and lead to human exposure by ingestion. The present study explored ways to obtain nanoplastic particles from real-life food containers. The first set of experiments gave rise to polypropylene nanoplastic suspensions with a hydrodynamic particle size range between 100 and 600 nm, whereas the same grinding process of polyethylene terephthalate (PET) produced suspensions of particles with a primary size between 100 and 300 nm. The exposure did not cause cytotoxicity measured by the lactate dehydrogenase (LDH) and water soluble tetrazolium 1 (WST-1) assays in Caco-2 and HepG2 cells. Nanoplastics of transparent PET food containers produced a modest concentration-dependent increase in DNA strand breaks, measured by the alkaline comet assay [net induction of 0.28 lesions/10^6^ bp at the highest concentration (95% CI: 0.04; 0.51 lesions/10^6^ base pair)]. The exposure to nanoplastics from transparent polypropylene food containers was also positively associated with DNA strand breaks [i.e., net induction of 0.10 lesions/10^6^ base pair (95% CI: −0.04; 0.23 lesions/10^6^ base pair)] at the highest concentration. Nanoplastics from grinding of black colored PET food containers demonstrated no effect on HepG2 and Caco-2 cells in terms of cytotoxicity, reactive oxygen species production or changes in cell cycle distribution. The net induction of DNA strand breaks was 0.43 lesions/10^6^ bp (95% CI: 0.09; 0.78 lesions/10^6^ bp) at the highest concentration of nanoplastics from black PET food containers. Collectively, the results indicate that exposure to nanoplastics from real-life consumer products can cause genotoxicity in cell cultures.

## Introduction

The impact of plastic litter on the ecological system has been a matter of concern for decades, although potential hazards to humans until recently appears only to have evoked modest attention. One reason could be that particle toxicologists have focused on health effects related to air pollution and particles that are smaller than 100 nm in diameter (i.e., so-called nanomaterials). The toxicology of nanomaterials has been highly useful for the understanding of ultrafine air pollution particles (1). The diameter of microplastics are between 1,000 nm and 5 μm in diameter, whereas nanoplastics are smaller than 1,000 nm.

Studies on stool samples have documented that humans are exposed to microplastic via the gastrointestinal tract (2). Infants appear to have higher exposure to microplastics than adults do (3). Consumption of microplastic via diet is an important source of particles in adults (4). House dust has been shown to be an important source of microplastics for children (5). It has also been shown that synthetic polymers and fibers are important airborne microplastics in dwellings (6). Microplastics have been detected in human lung (7) and placenta tissue (8).

One detrimental outcome of particle exposure is genotoxicity, which may be a direct consequence of interaction between particles and DNA or an indirect effect related to oxidative stress and inflammation (9). The single cell gel electrophoresis (comet) assay has been used in many cell culture studies, animal experiments and human exposure studies on both air pollution particles and nanomaterials (10). Similarly, the comet assay has been used extensively in ecotoxicology, using various sentinel species (11, 12). Interestingly, the research on genotoxic effects of plastic particles is much more developed in ecotoxicology as compared to human toxicology. Table 1 summarizes results from a survey of plastic particles in various studies on DNA damage measured by the comet assay in marine and terrestrial animals. Collectively, the studies indicate that the majority of studies have used standard particles such as polystyrene (23 studies) and polyethylene (6 studies), whereas only few studies have investigated the effect of particles from “real-life” plastic items. The latter group encompasses a study on microplastics from items collected from grinded cutlery, litter on sandy beaches and tumble dryer lint. These studies have shown genotoxicity in animal species after exposure to microplastic particles (13–15). To the best of our knowledge, there are no studies on genotoxic effects of real-life plastic debris particles in human cells (38). However, recent studies have used the comet assay to assess DNA strand breaks and oxidatively damaged DNA on human cells after exposure to either polyethylene or polystyrene (39–41).

**Table 1:** DNA damage measured by the comet assay in marine or terrestrial species after exposure to plastic particles^a^.

**Sample**	**Species**	**Number of exposure groups (time)**	**Effect**	**References**
Tumble dryer lint (fibers)	Mussels (*Mutilus galloprovinciallis*); hemocytes	3 (7 days)	Increased at highest (25%) and middle (17%) exposure level compared to control (2%)	(13)
Cutlery (PS, 65–125 μm, grinded)	Earthworm (*Eisenia fetida*); coelomocytes	3 (28 days)	Increase (concentration-dependent: 10, 15, 25 vs. 2% in controls)	(14)
Microplastic from sandy beaches (Hawaii) [consisting of 27% PE, 72% PP and 1% PS], which were grinded and filtered (600 μm, D50 = 305 μm)^b^	Japanese medaka larvae	3 (14 days)	Increased in lowest (8%) and middle (5%) exposure groups compared to controls (2%)	(15)
Microplastic from sandy beaches (Eastern Islands), which were grinded and filtered [consisting of 94% PE, 6% PP and 1% PS] (316 μm)^b^	Japanese medaka larvae	3 (14 days)	Unaltered (22%) compared to control (22%)^c^	(15)
Microplastic mixture [40% LDPE, 25% HDPE, 25% PP, 10% PS] (<600 μm; D50 = 409 μm)^b^	Japanese medaka larvae	1 (14 days)	Unaltered (4 vs. 2% in controls)	(15)
PE (<100 μm)	Mussels (*Mutilus galloprovinciallis*); hemocytes	1 (7 days)	Increase (30 vs. 10% in control)	(16)
PE (300 μm) [in cadmium-contaminated soil]	Earthworm (*Eisenia fetida*); sperm	4 (28 days)	Increased (4.5, 4.0, 6.5, and 8.5 vs. 2% in controls)	(17)
PE (with TiO_2_, 10–90 μm)	Neotropical teleost (*Prochilodus lineatus*); erythrocytes, gill and hepatic cells	1 (24 and 96 h)	Increased in erythrocytes (180 vs. 80 a.u.) at 96 h. Increased in liver at 24 h (80 vs. 20 a.u.) and 96 h (125 vs. 50 a.u.). No effect in gill cells	(18)
LDPE (11–13 μm)	Clamps (*Scrobicularia plana*); hemocytes	1 (14 days)	No effect (results reported as tail length and tail moment)	(19)
LDPE (11–13 μm)	Clamps (*Scrobicularia plana*); hemocytes	1 (14 days)	No effect (results reported as tail length and tail moment)	(20)
LDPE (20–25 μm)	Mussels (*Mutilus galloprovinciallis*); hemocytes	1 (7, 14, or 28 days)	No effect (22%, 37% in exposed and unexposed at 7 and 14 days; 30% vs. 25% at day 28 in exposed and controls, respectively)	(21)
PS (<100 μm)	Mussels (*Mutilus galloprovinciallis*); hemocytes	1 (7 days)	Increase (22 vs. 10% in control)	(16)
PS (110 nm)	Mussels (*Mutilus galloprovinciallis*); hemocytes	5 (4 days)	Increased at three highest concentrations (maximally 40% increased as compared to controls)	(22)
PS (0.5 μm)	Mussels (*Mutilus galloprovinciallis*); hemocytes	1 (7 and 26 days)	Increase (40 vs. 25% in control) after 26 days. No effect after 7 days	(23)
PS (4.5 μm)	Mussels (*Mutilus galloprovinciallis*); hemocytes	1 (7 and 26 days)	Increase (40 vs.25% in control) after 26 days. No effect after 7 days	(23)
PS (55 nm)	Zebrafish (*Danio rerio*); blood cells	1 (1, 3, or 5 days)^d^	Increase (15 vs. 7% in controls)	(24)
PS (100 nm)	Zebrafish (*Danio rerio*); blood cells	1 (1, 3, or 5 days)^d^	Increase (12 vs. 7% in controls)	(24)
PS (5–12 μm)	Zebrafish (*Danio rerio*); gill cells or liver)	1 (21 days)	Increase in gill cells (20 vs. 2%) and liver (21 vs. 1%)^e^	(25)
PS (23 nm)	Grass carp (*Ctenopharyngodon idella*); blood	1 (3 days)	Increase (3 vs. 1% in controls)	(26)
PS (23 nm)	Grass carp (*Ctenopharyngodon idella*); blood	3 (20 days)	Increase (18, 28, and 38% vs. 3% in controls)	(27)
PS (100 nm)	Earthworm (*Eisenia fetida*); coelomocytes	2 (14 days)	Increase (10 and 20%) at both exposure levels compared to controls (7%)	(28)
PS (1.3 μm)	Earthworm (*Eisenia fetida*); coelomocytes	2 (14 days)	Increase (16 and 22%) at both exposure levels compared to controls (7%)	(28)
PS (100 nm)	Earthworm (*Eisenia fetida*); coelomocytes	1 (21 days)	Increase (8 vs. 6% in controls)	(29)
PS (1 μm)	Earthworm (*Eisenia fetida*); coelomocytes	1 (21 days)	Increase (7 vs. 6% in controls)	(29)
PS (10 μm)	Earthworm (*Eisenia fetida*); coelomocytes	1 (21 days)	Increase (12 vs.6% in controls)	(29)
PS (100 μm)	Earthworm (*Eisenia fetida*); coelomocytes	1 (21 days)	Increase (11 vs. 6% in controls)	(29)
PS (65–125 μm)	Earthworm (*Eisenia fetida*); coelomocytes	3 (28 days)	Increase (concentration-dependent: 12, 20, 33 vs. 2% in controls)	(14)
PS (0.5 μm)	Clamp (*Tegillorca granosa*); hemocytes	1 (14 days)	Increase (reported as degree of DNA damage)	(30)
PS (30 μm)	Clamp (*Tegillorca granosa*); hemocytes	1 (14 days)	No effect (reported as degree of DNA damage)	(31)
PS (30 μm)	Clamp (*Tegillorca granosa*); hemocytes	1 (14 days)	No effect (reported as degree of DNA damage)	(32)
PS (20 μm)	Clamps (*Scrobicularia plana*); hemocytes	1 (14 days + 7 days depuration)	Increase (17 vs. 14% in controls)	(33)
PS (220 nm)	Gill and intestinal epithelial cell lines from rainbow trout	1 (48 h)	Unaltered in gill (3 vs. 1%) and intestinal epithelial cells (1 vs. 1% in controls)^f^	(34)
PS (8 μm)	Zebrafish (heart)	1 (21 days)	Increased (18 vs. 0.2% in controls)	(35)

a *The literature survey encompasses only pristine particles or debris from plastic litter. Studies were identified by search on PubMed using microplastic, nanoplastic and comet assay as terms. Additional studies were obtained from reference lists of the identified articles. The genotoxicity results are reported as percentage of the fluorescence in the comet tail (%) or arbitrary units (a.u.) as primary comet assay descriptors. Abbreviations are median diameter size (D50), low-density polyethylene (LDPE), polyethylene (PE), polypropylene (PP) and polystyrene (PS).*

b *The authors have also published an assessment of extractable organics from microplast samples (36, 37).*

c *Exposure to microplastics produced genotoxicity, measured by the Fpg-modified comet assay.*

d *Results are only reported from one time point, although which is not specified.*

e *Essentially the same results in Perch fluviatilis (gill cells: 22 vs. 2%, liver 24 vs. 1%).*

f *The study included the Fpg-modified comet assay (unaltered level of genotoxicity after particle exposure)*.

The aim of this study was to assess genotoxicity of plastic debris particles from real-life consumer products, namely food containers that were purchased in a local supermarket. There is no standardized procedure for degrading real-life plastics or minimal requirements for sufficient characterization of the particle suspension. The article describes results from experiments on ways to obtain particle suspensions from real-life plastic items. We have characterized the particle size in suspensions, whereas a complete chemical analysis of additives and other chemical compounds has not been done. There are approximately 7,000 additives used for the production of real-life plastic items, although it depends on type of polymer and expected use (42). We selected food containers made of polypropylene (PP) and polyethylene terephthalate (PET) to avoid types of plastics with potentially hazardous additives. The food containers were grinded with different blenders to plastic suspensions that was used to expose cells from the digestive system (i.e., Caco-2 cells) and the liver (i.e., HepG2 cells).

## Methods

### Preparation of Suspensions of Nanoplastic

The objective of the generation of nanoplastics was to set up a relatively fast method for grinding and subsequent isolation of small size particles in a harmless medium to human cells. The first part of the experiments used PP and PET plastic products (called “pilot study” in the article). The second part of the experiments focused on the production of PET plastic particles, where more effort was put into method development of the production part (called “main study” in the article).

**Figure 1:**
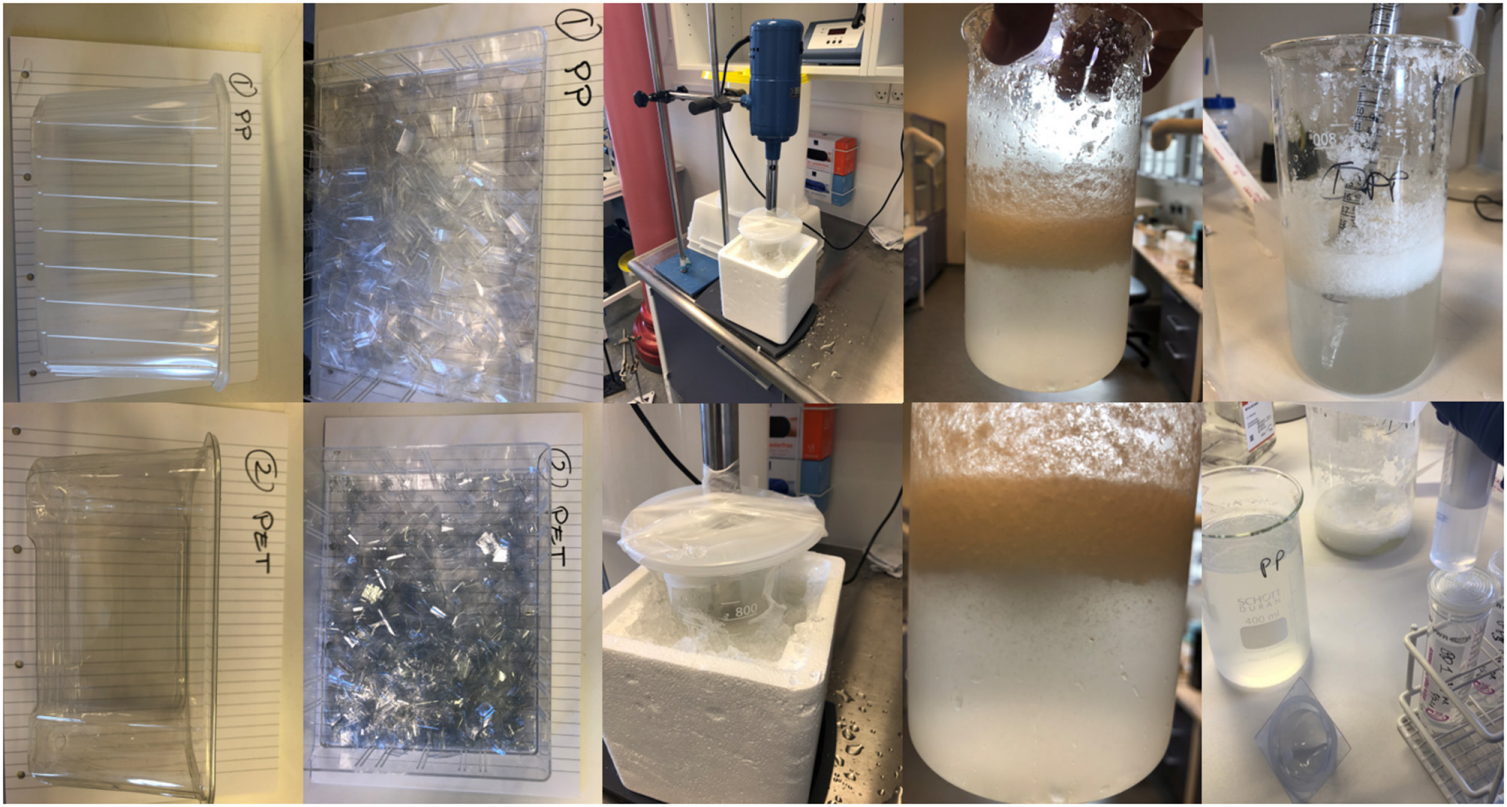
Preparation of polypropylene (PP) and polyethylene terephthalate (PET) suspensions for the pilot study. The images demonstrate from left to right the original food containers, pieces of containers, grinding process, primary slurry of PP and PET plastics particles.

For the pilot study, transparent plastic food containers of either PP or PET from a Danish supermarket were used. We used food containers because they were easy to obtain and relatively thin (i.e., They were easy to break and subsequently grind). Food containers are found as litter and might degrade to nanoplastics in the environment, whereas it seems unlikely that food containers release large amounts of nanoplastics and contaminate the food. PP and PET were chosen because they seemed to be the most common types of plastics for food containers, commonly found in environmental samples, and we considered them easier to grind than softer types of plastics such as polyvinyl chloride. They were washed with ethanol and left to dry. Afterwards the containers were cut into smaller pieces with a scissor. The small pieces in sterile isotonic water were grinded using an ultra-turrax T-45 blender for approximately 10 min on ice. This produced a slurry of large particles and hazy suspension of smaller particles that was extracted with a pipette. Afterwards the suspensions were filtered with a 0.45 μm sterile filter. The filtered suspensions were left to air dry on a heating block at 60–70°C in a LAF bench for approximately 5 days until the water had evaporated. Figure 1 shows images of steps in preparation of nanoplastics from transparent PP and PET food containers.

**Figure 2:**
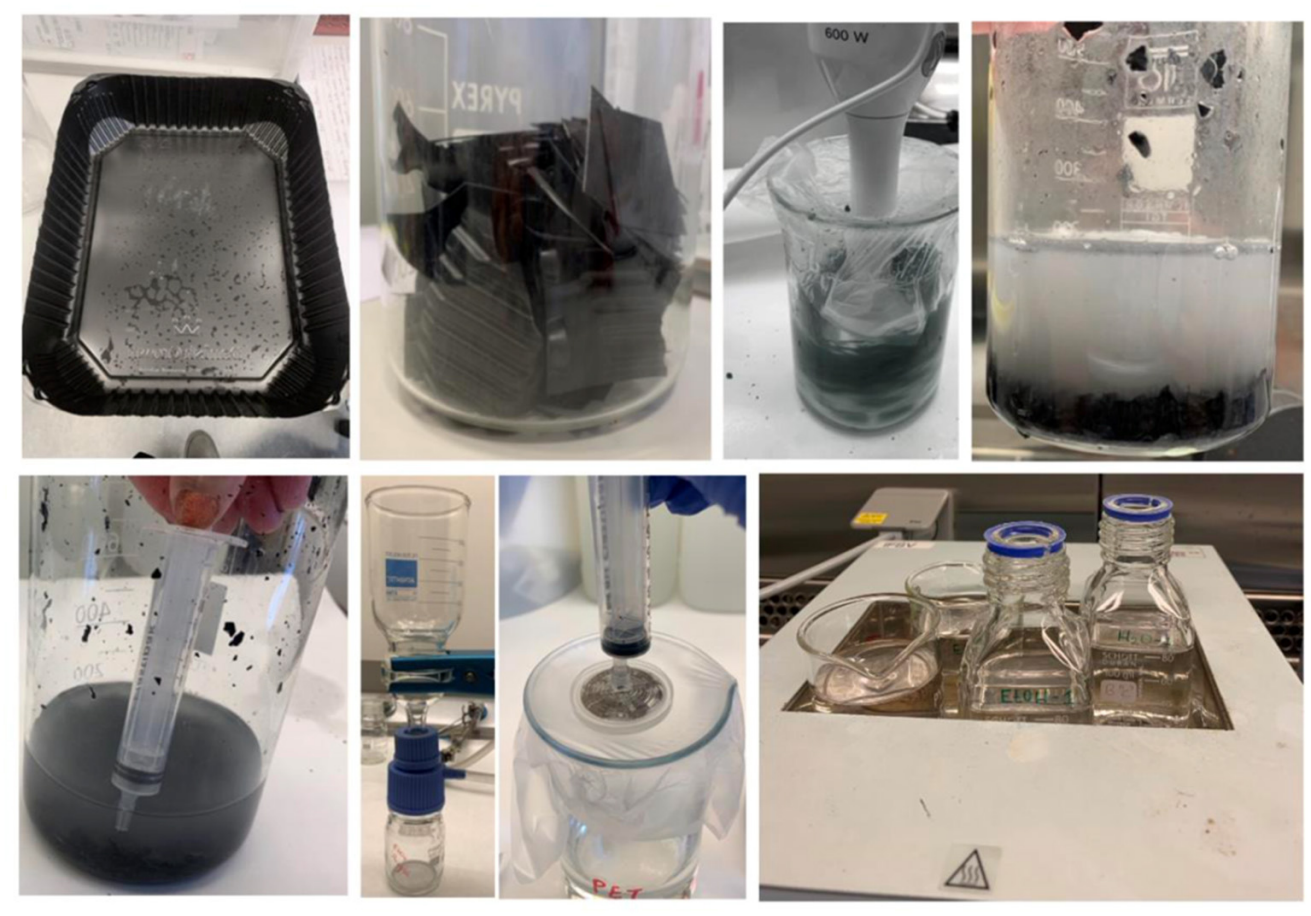
Preparation of PET particles for the main study. Top from left to right: the PET food container was first sterilized using 96% ethanol. The plastic was then cut into 5 cm long strips. The pieces were mixed with ethanol for 10 min at room temperature. The plastic-ethanol suspension was left to sediment for 5 min and 400 ml of the suspension extracted using a plastic syringe and filtered through a paper filter using a vacuum pump. The filtrate was further filtered through a 0.45 disk filter. The final suspension was left to evaporate at 65°C in a heating block.

For the second part of the study, we chose to use only PET material and selected raw meat containers from a Danish supermarket. Figure 2 shows images of steps in preparation of nanoplastics from black PET food containers. The containers contained a minimum of 80% recycled PET material. For the production, 100 g of pre-cut PET was suspended in 800 ml of 96% ethanol for 10 min at room temperature and subsequently grinded using an immersion blender (BOSCH MSM66020). The suspension was left to sediment for 5 min and 400 ml was extracted using a plastic syringe. The suspension was further filtered using a Whatman filter and finally run through a 0.45 μm sterile filter. The filtered solution was placed in a pre-weighted glass beaker and left to evaporate on a heat block at approximately 65°C. Two control beakers containing ethanol were also placed on the heat blocks and treated similarly to the PET suspension. The procedure was repeated three times adding three layers of particles and the beakers were weighed after each addition. The average weight change of the control beakers was estimated and subtracted from the weight of the beaker containing PET. Using a cell scraper, the PET particles were detached from the bottom of the beaker and placed into an Eppendorf tube and sterile water for injection (Gibco® Water for Injection for Cell Culture) was added to the PET to create a concentration of 10 mg/ml. The suspension was further filtered through a 0.45 μm sterile filter and sonicated in a water bath for 1 h, resulting in a stock suspension in water for injection that was diluted in cell media before exposure.

In the present study, we used the highest concentrations possible from the nanoplastic slurries in the cell culture experiments. As such, the higher concentration in the second trial is due to refinement of the plastic grinding and isolation procedure, which made it possible to obtain higher concentrations. It is not possible to compare the concentrations in the present study to the actual exposure in the gastrointestinal tract, because the magnitude of nanoplastic ingestion in humans is uncertain. In general, it is uncertain if the biological response to the same concentration of particles *in vitro* and human tissue is comparable as cultured cells are maintained in a non-physiological environment in the culture flasks and there is a selection of robust cells during the isolation from the original tissue (43).

#### Hydrodynamic Particle Size

The hydrodynamic particle size of the final plastic suspensions was analyzed by Nanoparticle Tracking Analysis (NTA) (NanoSight LM20). We have previously demonstrated that similar estimates of the hydrodynamic particle size is obtained with NTA and dynamic light scattering on suspensions of latex beads (100 nm), nanosized carbon black (Printex 90), and nanosized and fine titanium dioxide (44). We used isotonic saline for the hydrodynamic particle size measurements because there is variation in background levels of particles in full cell culture medium (45, 46), which we suspect originates from batch variation in FBS. In the present study, the background level of particles (and their sizes) were high in minimal essential medium (MEM, Sigma, Cat no. M2279) as compared to isotonic saline (results not shown). Previous studies on particles from combustion of fossil diesel, biodiesel, candle lights as well as urban dust (standard reference material 1649b and food-grade titanium (E171) have not revealed a systematic difference in particle sizes of suspensions in isotonic saline and cell culture medium (45, 47–51).

The analysis was performed in filtered water for injection as the cell medium with serum contained a high background in the NanoSight measurements. Five 1-min videos were recorded and analyzed by the NanoSight optical tracking system 3.0. The analysis determines the size distribution and the particle number concentration, from which the mass concentration was calculated by using a density of 1.38 and 0.9 g/cm^3^ for PET and PP, respectively. The PET suspension (5 μg/ml) was tested for presence of endotoxins with the Limulus Amebocyte Lysate (LAL) Pyrogent kit (Lonza E194-06), using a standard curve with *Escherichia coli* endotoxin O55:B5 (Lonza Material number: 7360, Batch no: 0000378664) (0.03, 0.06, 0.125, 0.25, and 0.5 EU/ml). No presence of endotoxins was detected in the particle suspension. Endotoxin-spiked samples (0.25, 0.125, 0.0625 EU/ml) showed unequivocal clot formation at 0.25 EU/ml, which was higher than the threshold of clot formation in the standard curve (i.e., 0.125 EU/ml) suggesting a slight inhibition of the biological response of endotoxin by nanoplastics.

#### Cell Culture

Human hepatocellular carcinoma (HepG2) and colorectal adenocarcinoma epithelial (Caco-2) cell lines were obtained from the American Type Culture Collection (Manassas, VA, USA). HepG2 and Caco-2 cells are widely used in toxicology, including studies on the comet assay (52, 53). Both cell types can internalize particles such as polystyrene nanoplastics (41, 54, 55). In addition, we use these cells because of the high biosafety level (i.e., virus-transformed cells have typically biosafety classification that warrants special laboratory facilities to culture the cells). The cells were cultured in Eagle's Minimum Essential Medium supplemented with 10% fetal bovine serum (FBS), 1% L-glutamine and essential amino acids, and 1% penicillin/streptomycin (all from Gibco). Incubations were carried out at 37°C in an atmosphere of 5% CO_2_. All experiments were performed on cells within passage 4–25.

#### Cytotoxicity (WST-1 and LDH Assays)

We have used water soluble tetrazolium 1 (WST-1) and lactate dehydrogenase (LDH) assays because they detect different mechanisms of action of cytotoxicity (i.e., cell membrane damage and metabolic activity). The cells were exposed to nanoplastics for 24 h to increase sensitivity of the cytotoxicity assays and avoid false positive comet assay results due to dying cells, which may not be detected after 3 h exposure. Three technical replicates in each exposure group of HepG2 and Caco-2 cells were seeded into a 96-well-plate (50,000/well), and the next day the cells were exposed to particle suspensions. Medium without particles was used as concurrent negative control. Cells exposed to cell medium with 1% Triton X-100 (Sigma-Aldrich, USA) served as positive control. After 24 h of exposure, the cell medium with particles was removed (and saved for the LDH assay), and the WST-1 reagent (Roche Diagnostics GmbH, Mannheim, Germany) in fresh cell medium (10% in 100 μl medium) was added to each well. The plate was incubated for 2 h before the absorbance was measured with a spectrophotometer (Multiskan Ascent, USA) at a wavelength of 450 nm with 630 nm as reference wavelength.

The LDH assay was performed in the same experimental setup as the WST-1 assay. The LDH activity measurement was performed with the Cytotoxicity Detection Kit (Lactate Dehydrogenase Activity, Roche Diagnostics GmbH, Germany) according to the manufacturer's protocol. The LDH working solution was added to the supernatant from the cell culture and left to incubate for 30 min before analysis with a spectrophotometer. The absorbance was measured at a wavelength of 500 nm with 630 nm as reference wavelength.

#### Cell Cycle Distribution

HepG2 or Caco-2 cells were seeded in a 6-well plate overnight (500,000 cells per well). The next day the cells were exposed to particles from grinded black colored PET food containers for 24 h at 37°C and 5% CO_2_. Serum free cell medium (SFM) was used as control for impeded progression of the cell cycle (i.e., positive control). Following the exposure, medium was removed, and the cells were washed with 1 ml of phosphate buffered saline (PBS) with 2% bovine serum albumin (BSA) and harvested using 350 μl of 0.05% trypsin-EDTA and 650 μl of cell medium. The cells were subsequently centrifuged for 5 min at 500 g, the supernatant was removed, and the cell pellet was resuspended in the small amount of remaining supernatant. The resuspended cells were fixed using 1.3 ml of cold methanol and incubated for 24 h at −20°C for the HepG2 cells and 1.3 ml of cold ethanol incubated for 2 h at −20°C for the Caco-2 cells. Following the incubation, the cells were centrifuged for 5 min at 500 g, and the supernatant was discarded. The cell pellet was resuspended and the cells were washed with 1 ml of PBS with 2% BSA and centrifuged for 5 min at 500 g, and the supernatant removed. The cell pellet was resuspended in 500 μl of FXCycle^TM^ (Thermo Fisher Scientific) staining solution containing 50 μg/ml propidium iodide and 100 μg/ml RNAse A in PBS and incubated for 30 min in 37°C and 5% CO_2_.

The stained cells were analyzed using a BD Accuri C6 flow cytometer with excitation at 488 nm. Forward scatter (FSC) and side scatter (SSC) were used to record information on cell size and granularity (complexity) of the cells, respectively. For each sample, 5 × 10^4^ cells were counted in a gate excluding cell debris and dead cells. A second gating was used to only include single cells in a plot of FSC-H vs. FSC-A. The cell count vs. fluorescence (propidium iodide staining) were used to assign cell cycle phases accordingly.

#### Intracellular ROS Production

HepG2 or Caco-2 cells were seeded overnight in a black bottomed 96-well plate at a density of 50,000 cells per well. The next day the cells were incubated for 15 min with 10 μM of 2'7'-dichloroflourescin diacetate (CAS no. 4091-99-0, Sigma-Aldrich, Cat no. D6883) in Hank's balanced salt solution (HBSS, Gibco) at 37°C and 5% CO_2_. After the incubation with the probe, the cells were washed with HBSS to remove extracellular probe and subsequently exposed to 200 μl of the microplastic suspension. H_2_O_2_ (CAS No. 7722-84, Sigma-Aldrich, Lot. 18A164128, 1 mM for HepG2 cells and 500 μM for Caco-2 cells) was used as positive control. The H_2_O_2_ concentration differed because HepG2 cells appeared to be less sensitive than Caco-2 cells in preliminary experiments. Exposures were carried out in technical triplicates on 3 different days. The cells were incubated for 3 h at 37°C and 5% CO_2_. After the incubation, the cells were washed twice with HBSS and finally resuspended in 100 μl of HBSS. Fluorescence was measured at 490 nm emission and 520 nm excitation.

#### DNA Damage Analysis by the Comet Assay

The comet assay is widely used in particle toxicology to detect DNA damage by exposure to a range of nanoparticles as well as complex mixtures such as combustion or air pollution. The standard comet assay—like the alkaline elution and alkaline unwinding assays—detects DNA strand breaks or lesions that are converted to breaks by the alkaline condition (56). It is well-known that procedures affects the characteristics of comets (e.g., the comet tail length is proportional to the electrophoresis time) (57). Thus, comet images or primary comet descriptors (e.g., percent tail DNA) are not equivalent to the actual number of DNA lesions. By using calibration with ionizing radiation, it is possible to obtain information on the actual number of lesions in DNA from primary comet descriptors (58). Using this conversion of primary comet descriptors to actual numbers of lesions in DNA, the comet assay has been validated against chromatographic assays for detection of oxidatively damaged DNA (59–61). The European Comet Assay Validation Group conducted a number of ring-trials with the aim of assessing the inter-laboratory variation procedures and results by the comet assay (62–68). Lately, the hCOMET project are conducting ring-trials to test potassium bromate as a positive assay control in cryopreserved samples (69, 70). The comet assay has been thoroughly validated in studies that led up to the adoption of the technique for OECD guideline test no 489 (71–73). Lastly, it has been shown that the comet assay has reasonable sensitivity (79%) and specificity (76%) in multi-organ tests on rodents (74, 75). In addition, recent pooled analysis from human biomonitoring studies have demonstrated that high levels of DNA strand breaks in blood cells predicts risk of death (76, 77).

HepG2 or Caco-2 cells were seeded overnight in a 24-well plate at a density of 250,000 cells per well. On the day of experiment, the cell culture medium was removed and medium with nanoplastics or H_2_O_2_ (100 μM, positive control) was added. The cells were subsequently exposed at 37°C and 5% CO_2_ for 3 h (nanoplastics) or 15 min (H_2_O_2_). The incubation period for H_2_O_2_ is short because it is a fast-acting oxidant and DNA strand breaks are repaired relatively fast after H_2_O_2_ exposure. Levels of DNA strand breaks were determined by the alkaline comet assay as previously described and reported according to the Minimum Information for Reporting on the Comet Assay (MIRCA) recommendations (78, 79). The exposure medium was removed and the cells were washed with PBS. Subsequently, 150 μl of trypsin was added and the cells were incubated 15 min, until the reaction was terminated by addition of 350 μl medium. Suspensions of cells (75 μl) were mixed with 600 μl of 0.75% agarose gel and 120 μl of this suspension was applied onto Gelbond films (Cambrex, Medinova Scientific A/S, Hellerup, Denmark). The gel-embedded cells were lysed overnight (2.5 M NaCl, 100 mM Na_2_EDTA, 10 mM Trizma base, pH = 10). The samples were afterwards placed in electrophoresis buffer (1 mM Na_2_EDTA, 300 mM NaOH, pH > 13.1) for 40 min, and the electrophoresis was subsequently run for 25 min at 300 mA and 20 V (0.83 V/cm; cathode to anode). The samples were placed in neutralization buffer (0.4 M Trizma base) for 15 min, followed by overnight treatment in 96% ethanol to preserve the embedded samples. The nuclei were stained with YOYO-1 dye (CAS No. 143413-85-8; 491/509 Thermo Fisher Scientific, Waltham, MA, USA) and scored manually under an Olympus CX40 fluorescence microscope at 40x magnification. The samples were blinded when scoring the comets, and the level of DNA damage was determined by using a five-class scoring system (arbitrary score range 0–400). For each sample, 100 randomly chosen nucleoids per slide were visually scored. The comet score was transformed to lesions per 10^6^ base pairs (bp) by the use of a calibration curve from the European Comet Assay Validation Group where one arbitrary unit (0–100 a.u. scale) corresponds to 0.0273 lesions/10^6^ base pairs as described previously (63).

#### Statistics

The results were analyzed by linear mixed effects model, and subsequently linear regression or ANOVA in separate strata of HepG2 and Caco-2 cells. The mixed effect linear regression model contained the cell type as categorical variable and concentration as continuous variable. Cell culture medium without nanoplastics was used as vehicle control. Statistical analyses were carried out in Stata 15 (StataCorp LCC, College Station, TX, USA). The results are reported as mean and standard error of the mean (SEM) of 2–3 independent experiments. Net inductions of DNA strand breaks and 95% confidence interval (95% CI) are reported to give an impression of the effect size and experimental variation.

## Results

### Hydrodynamic Particle Size of Nanoplastic Suspensions

**Figure 3:**
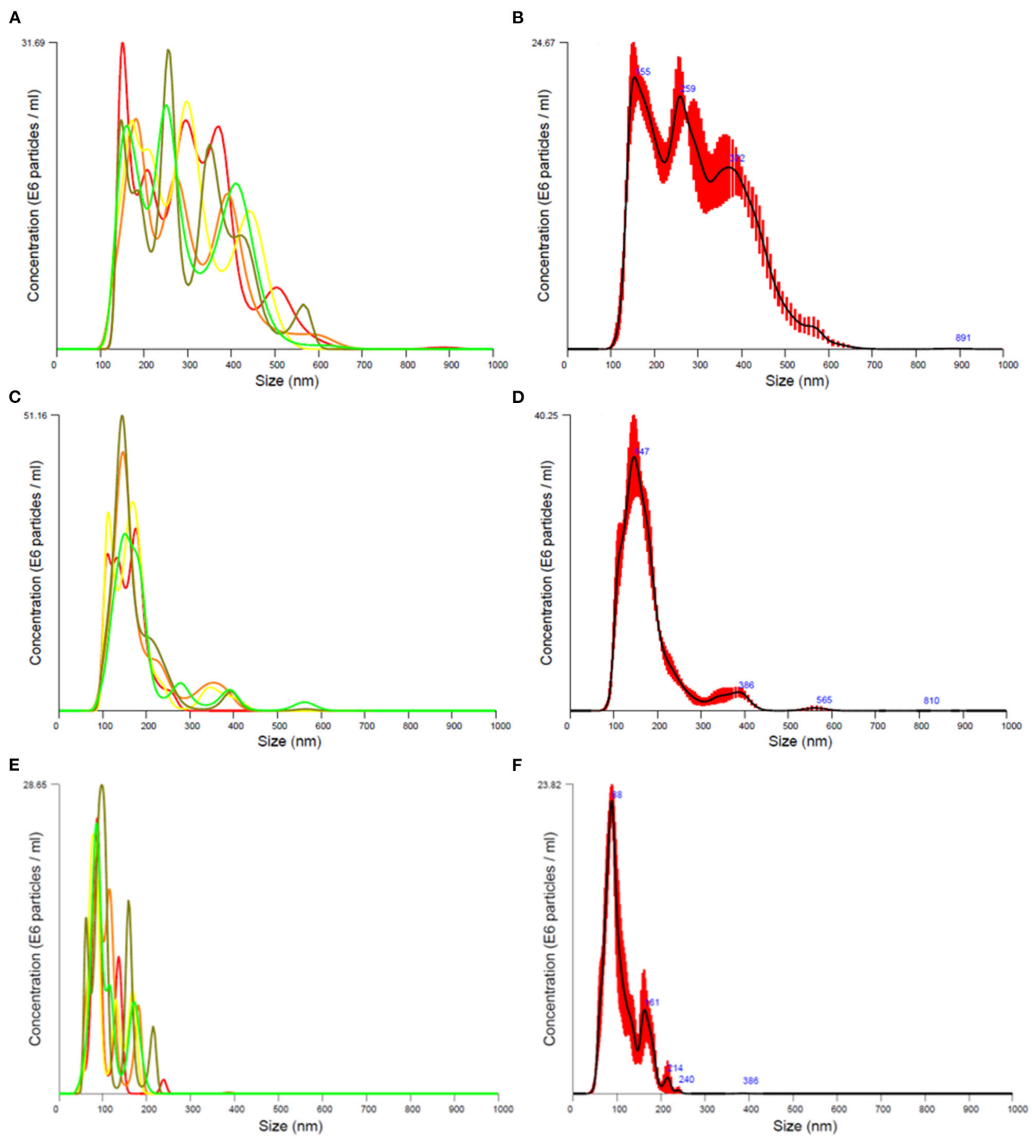
Particle size distribution of final exposure suspensions from transparent polypropylene **(A,B)** and polyethylene terephthalate plastics **(C,D)** in the pilot study, and suspensions from black polyethylene terephthalate plastics in the main **(E,F)** study. The suspensions were analyzed in filtered water for injection. The mean particle size distribution **(B,D,F)** has been obtained from five consecutive size distribution measurements **(A,C,E)**.

Particle size distributions of nanoplastic suspensions of PP and PET food containers are shown in Figure 3. The mechanical degradation of PP plastic food containers resulted in suspensions with a broad particle sizes distribution in two larger fractions, 80–250 nm and 200–600 nm. The suspension from transparent PET plastic food containers had a primary size distribution peak at 80–250 nm, and a lower peak of particles with particle sizes between 300 and 400 nm. The suspension from black PET containers contained mainly particles with diameters less than 240 nm. The particle number concentration was approximately 20 times lower than expected (for the main study) after particles were passed through a sterile filter (0.45 μm) in the end (Table 2). The mass concentration has been estimated (and reported in the article) on basis of the particle number concentration, mean particle size and density of the particles.

**Table 2:** Hydrodynamic particle size and concentration data from the Nanosight experiment^a^.

**Characteristic**	**PET (main study)**	**PET (pilot)**	**PP (pilot)**
Mean diameter (nm)	107 (10)	252 (100)	158 (55)
Particle number concentration (number/ml)	52 × 10^11^ (1.5 × 10^11^)	2.3 × 10^10^ (9.1 × 10^8^)	2.2 × 10^10^ (8.7 × 10^8^)
Stock suspension (mg/ml)	0.471 (0.14)	0.175	0.063
Final suspension (μg/ml)	0.6–7.1	0.001–0.063	0.003–0.175

a *Results are reported as mean and (standard deviation)*.

### Effects of Transparent PP and PET Particle Exposure in HepG2 and Caco-2 Cells

**Figure 4:**
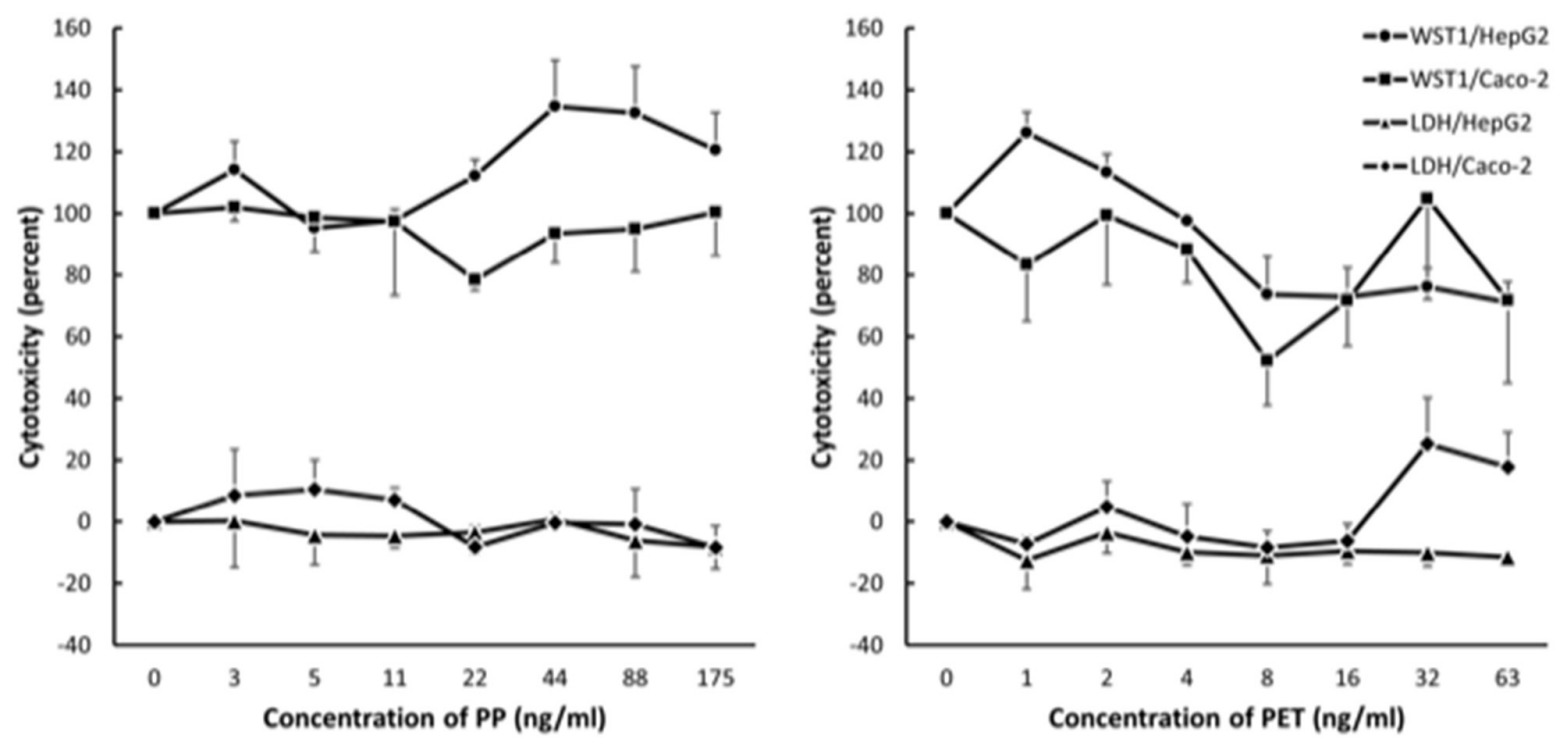
Cytotoxicity in Caco-2 and HepG2 cells after 24 h exposure to polypropylene (PP) and polyethylene terephthalate (PET). The results are reported as fold-difference compared to the positive control (LDH assay) and unexposed (WST-1 assay). Symbol and error bars are means and SEM from three independent experiments.

Results from the LDH and WST-1 experiments did not indicate cytotoxicity after 24 h exposure to PP in HepG2 and Caco-2 cells (Figure 4). The exposure to PET reduced the metabolic activity (WST-1 assay) in HepG2 cells (slope = −0.39 ± 0.12, *P* < 0.01, linear regression) and increased LDH leakage in Caco-2 cells (slope = 0.27 ± 0.10, *P* < 0.05, linear regression). Nevertheless, the cytotoxicity response was not consistent across cell types as mixed effect linear models were not statistically significant for PET (WST-1 assay: slope = −0.24 ± 0.12, *P* = 0.053; LDH assay: slope = 0.11 ± 0.06, *P* = 0.09).

**Figure 5:**
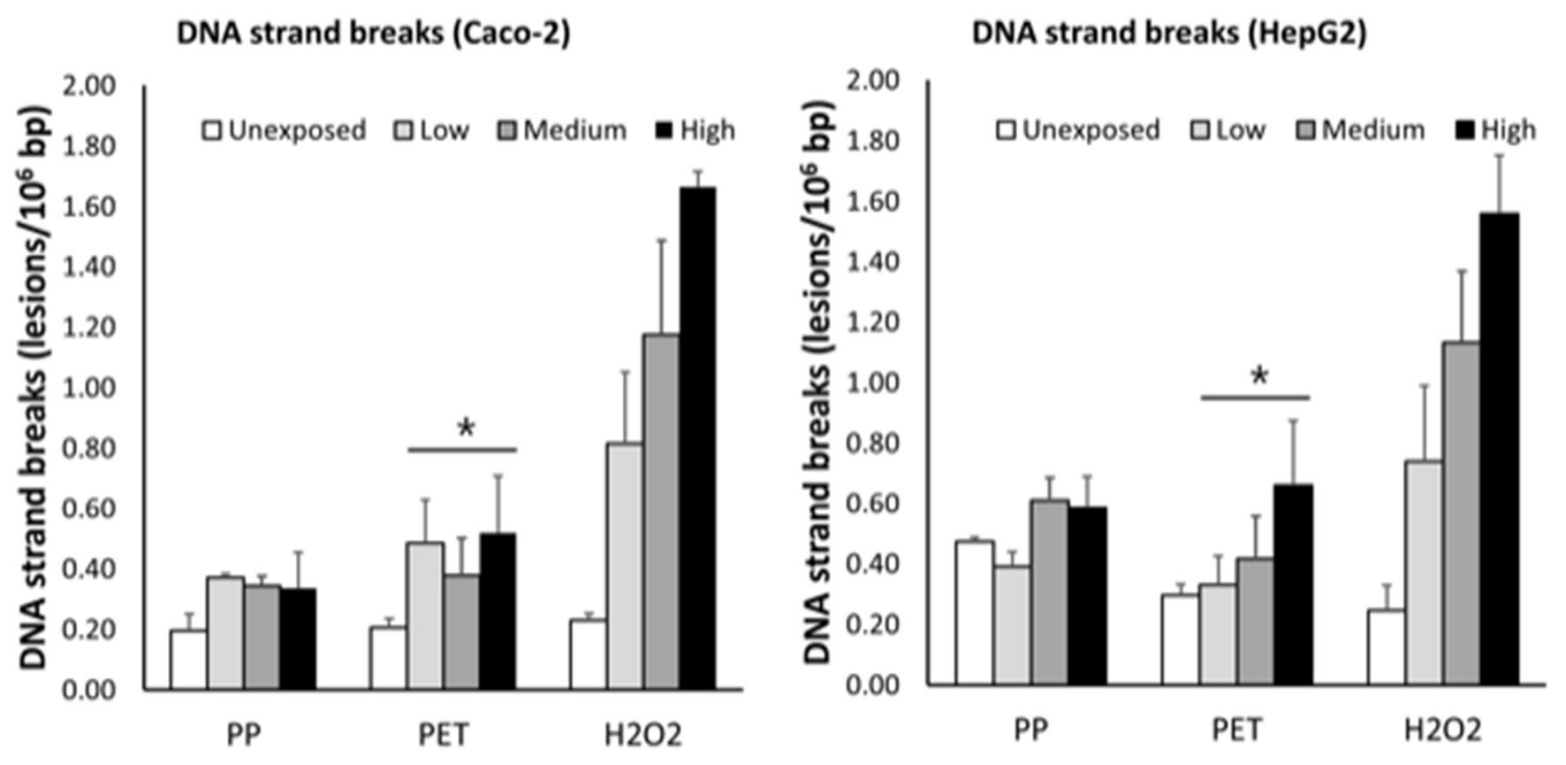
Levels of DNA strand breaks in Caco-2 and HepG2 cells after 3 h exposure to grinded particles of polypropylene (PP) and polyethylene terephthalate (PET) food containers. The high concentration is 63 ng/ml, 175 ng/ml, and 100 μM of PET, PP, and H_2_O_2_, respectively. The medium and low concentrations correspond to sequential two-fold dilutions. Each bar is the mean and SEM of three independent experiments, except H_2_O_2_ in Caco-2 cells (*n* = 2). *P < 0.05, linear mixed effect model.

Effects of PP and PET nanoplastic exposure on DNA strand breaks are shown in Figure 5. The exposure to PET was associated with a concentration-dependent increase in DNA strand breaks, which was not different in the two cell types (slope = 0.28 ± 0.11, *P* < 0.05; single-factor of “cell type”: *P* = 0.71). Based on this model, the highest concentration of PET generated 0.28 lesions/10^6^ bp (95% CI: 0.04; 0.51 lesions/10^6^ bp). However, the linear regression analyses and ANOVA in separate strata of HepG2 and Caco-2 cells did not indicate statistically significant effects. The exposure to PP was also positively associated with DNA strand breaks in mixed effects linear model, although this was not statistically significant (slope = 0.10 ± 0.06, *P* = 0.15). The induction of DNA strand breaks at the highest concentration was 0.10 lesions/10^6^ bp (95% CI: −0.04; 0.23 lesions/10^6^ bp). The positive control (H_2_O_2_) increased the level of DNA strand breaks in both HepG2 and Caco-2 cells in a concentration-dependent manner (*P* < 0.001, linear regression).

### Effect of Particles From Black Colored PET Food Container in HepG2 and Caco-2 Cells

**Figure 6:**
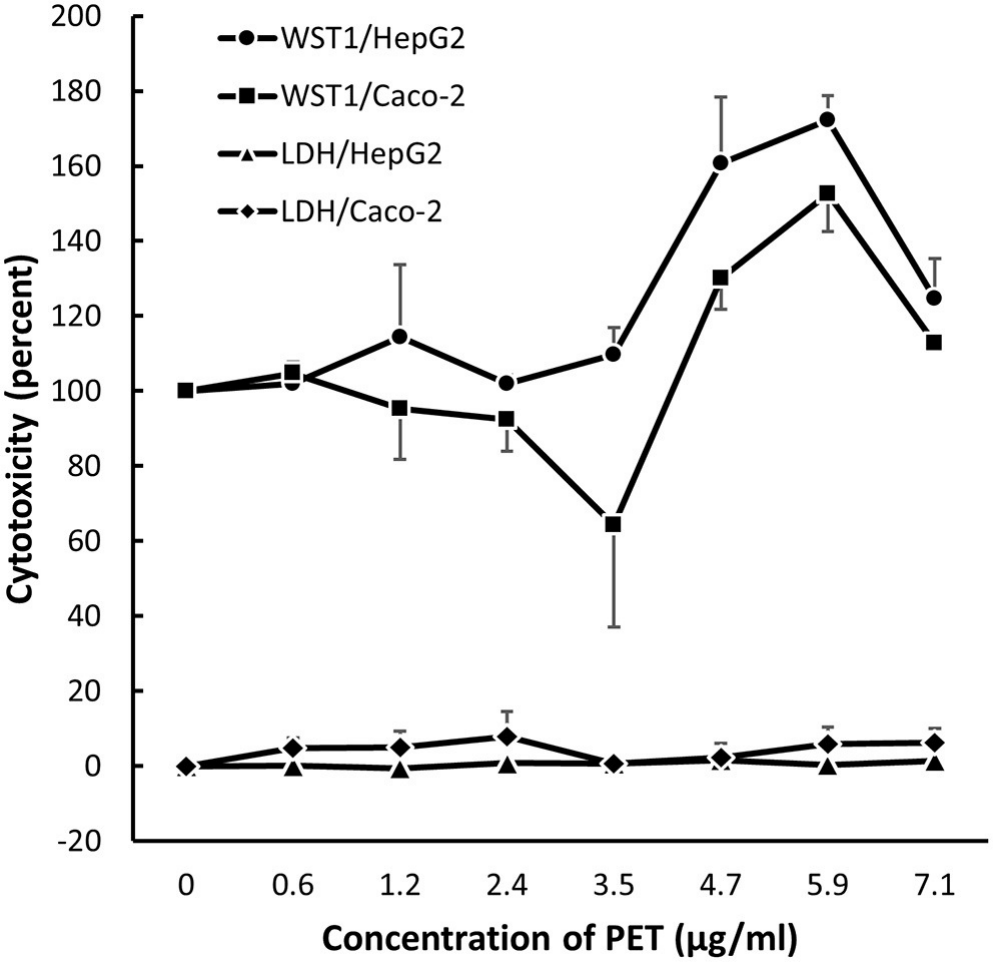
Metabolic activity (WST-1 assay) and cell membrane leakage (LDH assay) after 24 h exposure to nanoplastics from black PET food containers (main study). The results are mean and SEM from three independent experiments.

**Figure 7:**
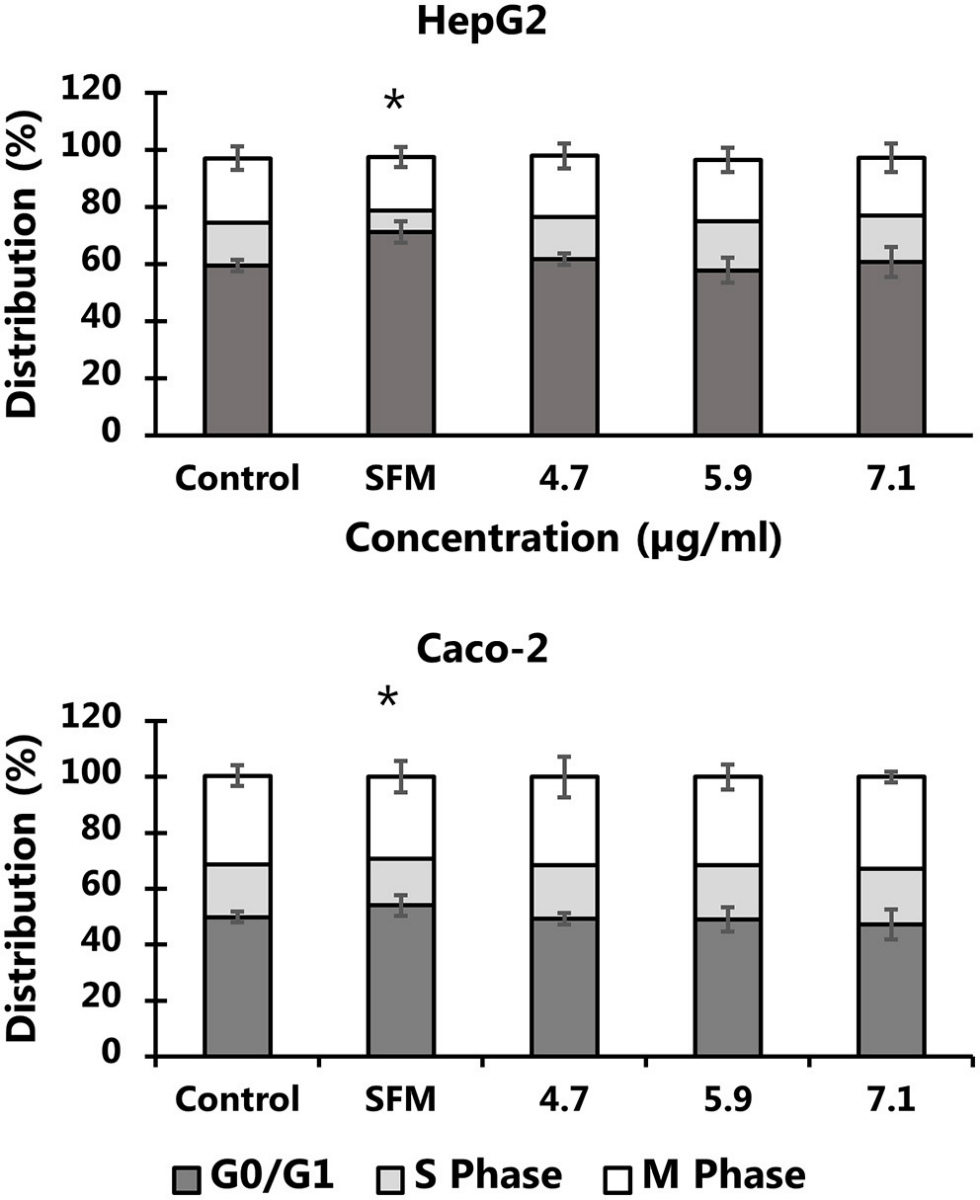
Cell cycle distribution HepG2 and Caco-2 cells after 24 h exposure to PET nanoplastics from black food containers. The results are means of 2-3 independent experiments (mean and standard deviation). The exposure to nanoplastics is not associated with changes in the cell cycle distribution (*P* > 0.05), whereas culture of cells in serum free medium (SFM) shifted the cell cycle to G0/G1 phase from DNA synthesis phase (S). *P < 0.05, linear mixed effects model.

**Figure 8:**
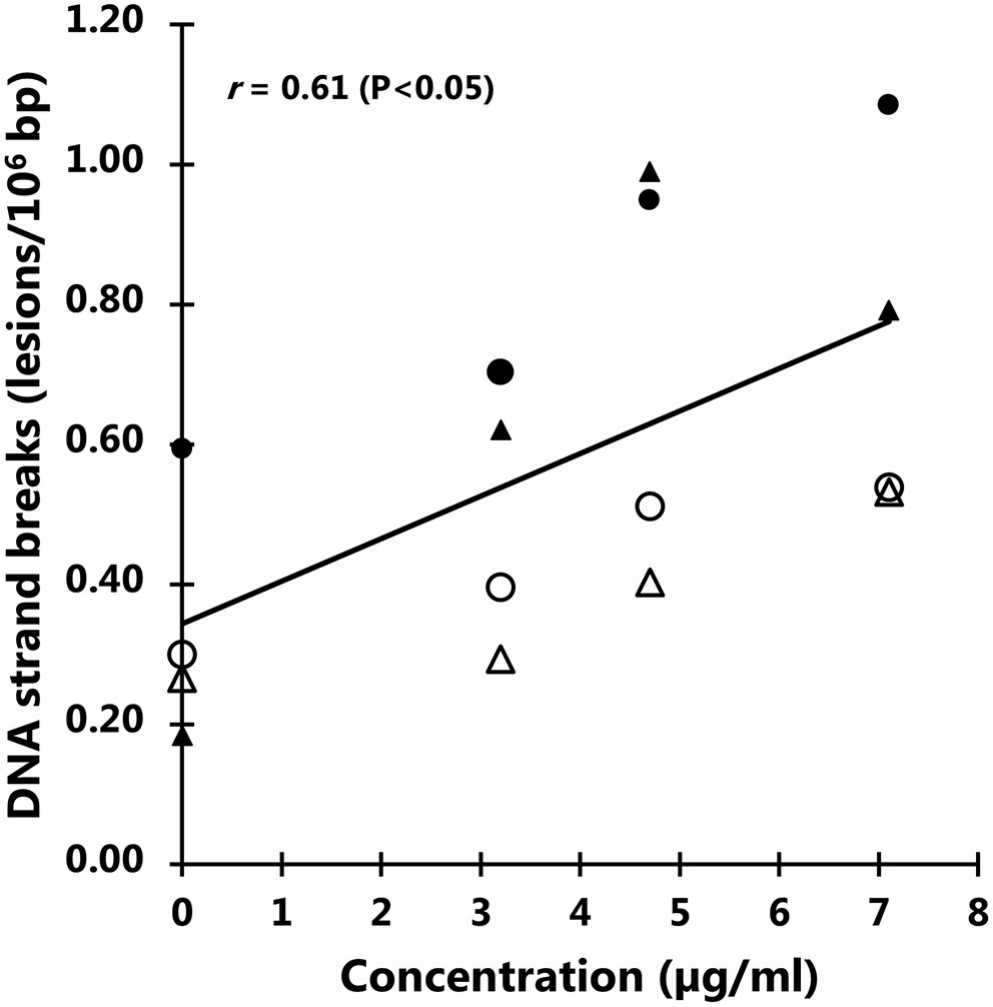
Levels of DNA strand breaks in Caco-2 and HepG2 cells after 3 h exposure to particles from black polyethylene terephthalate (PET) food containers. The correlation coefficient refers to the concentration response relationship in linear mixed effect model. Symbols are individual experiments. The positive control (100 μM H_2_O_2_) is 2.49 lesions/10^6^ bp (standard error of the mean = 0.06 lesions/10^6^ bp).

The exposure to particles from black colored PET food containers did not affect the level of cytotoxicity in cells (Figure 6) or distribution of cells in G0/G1, S or G2/M phases of the cell cycle (Figure 7). Cultures of HepG2 and Caco-2 cells in the positive control (SFM) has a slightly shifted cell cycle with more cells in G0/G1 phase (8.0%, 95% CI: 0.5%, 15%) and less cells in S phase (decline: 4.7%, 95% CI: 8.7%, 0.8%) and M phase (decline: 3.2%, not statistically significant). The exposure to PET nanoplastics did not affect the ROS production level in HepG2 and Caco-2 cells after 3 h exposure (Table 3), whereas the positive control (H_2_O_2_) was associated with a 2.2-fold increase in ROS production (*P* < 0.05, linear mixed effects model). The exposure to PET nanoplastics was associated with increased levels of DNA strand breaks in HepG2 and Caco-2 cells (*P* < 0.05, linear mixed effect model) and the concentration-response relationship was the same in the two cell types (Figure 8). The induction of DNA strand breaks at the highest concentration was 0.43 lesions/10^6^ bp (95% CI: 0.09; 0.78 lesions/10^6^ bp). The positive control (100 μM H_2_O_2_) generated very high levels of DNA strand breaks (2.49 ± 0.06 lesions/10^6^ bp). These comet assay experiments also included cryopreserved negative (0 μM H_2_O_2_) and positive (50 μM H_2_O_2_) controls of monocytic THP-1 cells. These assay controls did not indicate a difference between individual experiments (i.e., mean and standard deviation 0.08 ± 0.03 and 1.76 ± 0.18 lesions/10^6^ bp).

**Table 3:** Intracellular reactive oxygen species (ROS) production (relative to control) in HepG2 and Caco-2 cells after exposure for 3 h to nanoplastic from recycled PET food containers.

**Concentration (μg/ml)**	**HepG2**	**Caco-2**
0 (control)	1	1
0.1	1.02 ± 0.11	1.17 ± 0.11
0.2	1.22 ± 0.19	1.15 ± 0.09
0.4	1.14 ± 0.11	0.96 ± 0.16
0.9	0.80 ± 0.10	1.31 ± 0.22
1.8	0.94 ± 0.09	1.33 ± 0.21
3.6	1.43 ± 0.37	1.02 ± 0.26
7.1	1.04 ± 0.17	0.84 ± 0.19
Slope (± SEM)*	0.11 ± 0.20 (P=0.61)	−0.26 ± 0.19 (P=0.19)

* *Linear mixed effects model indicated no statistical significance of the nanoplastic exposure (Slope = −0.08 ± 0.14, P = 0.58). The positive control (H_2_O_2_) was associated with a 2.2-fold increase in ROS production (95% CI: 1.1, 5.3-fold) in linear mixed effects model (P < 0.05). Results are mean and SEM of three independent experiments*.

## Discussion

Relative few studies have assessed the hazards of real-life plastic debris particles on mammalian cells. A pioneering study, published in 2008, grinded a polymer fabric of polyethylene and PET in a freezer/mill and incorporated the material into standard rodent chow (80). Rats were exposed for 13 weeks and no toxicological effects relevant to the treatment were observed (80). The particle size of the microplastics was not determined in the original study, although a later study has estimated the particle size to 1–50 μm (average size 15–35 μm), based on the grinding procedure (81). A more recent study milled PP microplastics (25–200 μm in diameter) to particles with a size of 20 μm (50% of the particles were 5–10 μm) and showed modest effects in term of cytokine release and ROS production in human cell lines (82). Another study on real-life plastic used exposure to visible/ultraviolet light as environmental weathering process and showed that a disposable coffee cup lid was gradually degraded over a 56-day period to particles with an average size of 224 nm (83). We experimented with relatively low-tech procedures to grind plastic items and obtain suspensions in the size range of nanoplastics (i.e., less than 1,000 nm in average diameter). The size range in PP suspensions was 100–600 nm, whereas that of PET was 100–400 nm (pilot study) and <240 nm (main study). It has been shown that 4 mm PET plastics could be milled and sieved to suspensions of particles with sizes smaller than 200 μm and peak number distribution at approximately 150 nm (84). At least based on these examples, it appears applicable to obtain suspensions of nanoplastics, whereas it might be challenging to obtain nanoparticles (i.e., smaller than 100 nm) from plastic items.

There are notable differences between the studies using the comet assay in ecotoxicological studies and cells from humans (Table 1). The exposure time is much longer in non-human species (i.e., typically weeks in ecotoxicology as compared to hours-days in human toxicology). It is also interesting that the ecotoxicological studies indicate that even large polystyrene particles with diameters larger than 1 μm are genotoxic in the comet assay (14, 23, 25, 28, 29, 31, 33). On the contrary, studies on human cells have indicated very little effect on levels of DNA strand breaks measured by the comet assay. Domenech et al. did not observe genotoxicity in Caco-2/HT29 intestinal cells, with or without co-culture with Raji-B cells after exposure to nanosized polystyrene particles for 24 h (40). Likewise, Busch et al. reported unaltered levels of DNA strand breaks in co-cultures of intestinal cells after exposure to non-modified polystyrene and polyvinylchloride particles (85).

The statistical analysis of the comet assay results indicates a net increase between 0.10 and 0.27 lesions/10^6^ bp of PP and PET, respectively (pilot study). The concentrations and net induction of DNA strand breaks was slightly higher in the main study on recycled PET nanoplastics (i.e., 0.43 lesions/10^6^ bp). We have previously obtained statistically non-significant increases of approximately 0.23 DNA strand break/10^6^ bp in HepG2 and A549 cells after exposure to liposomes for 3 h (86). Studies on combustion-derived particles from our laboratory have typically demonstrated net increases of DNA strand breaks in the range of 0.2–0.8 lesions/10^6^ bp in cells after exposure to 100 μg/ml for 3–4 h (87–89). However, it should also be noted that we have used dispersion protocols with high-energy sonication that favors stable particle suspensions, whereas the sedimentation rate of particles might be lower. Using carbon-based nanomaterials, we have observed that approximately 10% of the administered dose deposits on cells at the bottom of the cell culture wells (90, 91). Longer incubation time increases the deposition, but it does not necessarily increase the level of DNA damage after particle exposure, which may be due to concurrent repair of DNA lesions. For instance, we have shown that 24-h exposure to carbon nanotubes increased the DNA repair activity in A549 cells (92).

It should be noted that the cell cycle distribution was unaltered after 24 h exposure, suggesting that the types of DNA lesions do not stall the replication. This suggests that the nanoplastics have produced relatively simple lesions such as single strand breaks and alkali-labile sites, whereas complex lesions (e.g., DNA cross-links or double strand breaks) have not been predominant. However, the unaltered ROS production in both HepG2 and Caco-2 cells suggests that the genotoxic mechanism of action is not oxidative stress. It is possibly a direct physical interaction between nanoplastic particles with DNA or replication machinery or leakage of chemicals from suspended nanoplastics are causing DNA damage. Nevertheless, we considered that food contact materials are included in stricter regulation due to safety concern than other types of plastics.

The present study has certain important limitations. The chemical composition of the plastic items has not been assessed. It can be speculated that hazardous chemicals have not been added to the plastic items that we have used because they are used for food products. As such it could be argued that the present study may underestimate the genotoxic effect of microplastics that are found in nature as they come from all sorts of plastic items. The physical characteristics (e.g., shapes, agglomeration, and sizes) of the nanoplastic suspensions have not been determined. These characteristics may have an influence on the biological activity of particles, although it—to the best of our knowledge—is uncertain if such characteristics systematically affects the genotoxic potential determined for instance by the comet assay. In addition, it should be noted that differences in physical characteristics has been a relevant issue in studies on well-defined nanomaterials. Research on real-life nanoplastics is somewhat similar to studies on air pollution particles; both are complex mixtures of particles and fibers with many different shapes, sizes, chemical constituents and agglomerates. Another uncertainty is the mass concentration, which was extrapolated from particle number concentrations. There is a risk of the exposure being in the low end of the concentration-response range, although it could also be argued that the concentrations in the present study are closer to realistic exposures than most other studies on particles that uses up to 10-fold or higher concentrations.

In conclusion, the present study shows that real-life plastic materials relatively easy can be degraded to nanoplastics by mechanical processes. Suspensions of these nanoplastics from PP and PET food containers generates genotoxicity in terms of DNA strand breaks measured by the comet assay, without cytotoxicity, ROS production or altered cell cycle distribution.

## Data Availability Statement

The original contributions presented in the study are included in the article/supplementary material, further inquiries can be directed to the corresponding author/s.

## Author Contributions

MH, IK, EM, and JS performed the experiments. MR and PM drafted the manuscript, which was approved by all authors. All authors contributed to the article and approved the submitted version.

## Funding

This study was supported by a grant from Carl og Ellen Hertz's Familielegat fund.

## Conflict of Interest

The authors declare that the research was conducted in the absence of any commercial or financial relationships that could be construed as a potential conflict of interest.

## Publisher's Note

All claims expressed in this article are solely those of the authors and do not necessarily represent those of their affiliated organizations, or those of the publisher, the editors and the reviewers. Any product that may be evaluated in this article, or claim that may be made by its manufacturer, is not guaranteed or endorsed by the publisher.
